# α-Linolenic Acid, A Nutraceutical with Pleiotropic Properties That Targets Endogenous Neuroprotective Pathways to Protect against Organophosphate Nerve Agent-Induced Neuropathology

**DOI:** 10.3390/molecules201119698

**Published:** 2015-11-12

**Authors:** Tetsade Piermartiri, Hongna Pan, Taiza H. Figueiredo, Ann M. Marini

**Affiliations:** 1 Molecular and Cellular Biology Graduate School Program, Uniformed Services University of the Health Sciences, 4301 Jones Bridge Road, Bethesda, MD 20814, USA; tetsade@yahoo.com.br; 2 Department of Neurology and Program in Neuroscience, Uniformed Services University of the Health Sciences, 4301 Jones Bridge Road, Bethesda, MD 20814, USA; hongna.pan@usuhs.edu; 3 Department of Anatomy, Physiology and Genetics, Uniformed Services University of the Health Sciences, 4301 Jones Bridge Road, Bethesda, MD 20814, USA; taiza.figueiredo.ctr@usuhs.edu

**Keywords:** α-linolenic acid, nutraceutical, natural product, rat, soman, neuroprotection, pleiotropic, BDNF, mTOR, neurorestoration

## Abstract

α-Linolenic acid (ALA) is a nutraceutical found in vegetable products such as flax and walnuts. The pleiotropic properties of ALA target endogenous neuroprotective and neurorestorative pathways in brain and involve the transcription factor nuclear factor kappa B (NF-κB), brain-derived neurotrophic factor (BDNF), a major neuroprotective protein in brain, and downstream signaling pathways likely mediated via activation of TrkB, the cognate receptor of BDNF. In this review, we discuss possible mechanisms of ALA efficacy against the highly toxic OP nerve agent soman. Organophosphate (OP) nerve agents are highly toxic chemical warfare agents and a threat to military and civilian populations. Once considered only for battlefield use, these agents are now used by terrorists to inflict mass casualties. OP nerve agents inhibit the critical enzyme acetylcholinesterase (AChE) that rapidly leads to a cholinergic crisis involving multiple organs. *Status epilepticus* results from the excessive accumulation of synaptic acetylcholine which in turn leads to the overactivation of muscarinic receptors; prolonged seizures cause the neuropathology and long-term consequences in survivors. Current countermeasures mitigate symptoms and signs as well as reduce brain damage, but must be given within minutes after exposure to OP nerve agents supporting interest in newer and more effective therapies. The pleiotropic properties of ALA result in a coordinated molecular and cellular program to restore neuronal networks and improve cognitive function in soman-exposed animals. Collectively, ALA should be brought to the clinic to treat the long-term consequences of nerve agents in survivors. ALA may be an effective therapy for other acute and chronic neurodegenerative disorders.

## 1. α-Linolenic Acid—An Essential Nutraceutical

α-Linolenic acid (ALA) is an essential omega-3 polyunsaturated fatty acid (PUFA) that is found in green leaves, seed oil (flax), pumpkin seeds, beans and walnuts; flaxseeds are the richest source of ALA [1]. ALA is an 18 carbon polyunsaturated fatty acid containing three double bonds at the 9, 12 and 15 positions. ALA plays an important role in brain function and protection as well as exhibiting anti-inflammatory and neuroplastic properties [2,3,4] and has a very wide safety margin [5,6]. ALA is a precursor of the long-chain PUFAs, eicosapentaenoic acid (EPA) and docosahexaenoic acid (DHA).

Early work showed that administration of ALA resulted in an increase of the omega-3 PUFAs 7,10,1,3,16,19-docosapentaenoic acid and 4,7,10,13,16,19-docosahexaenoic acid in the brain [7]. Humans and rats have the ability to metabolize ALA to form EPA and DHA, but the overall conversion appears to be limited in humans [8] and in rats [9]. In a single male subject, a significant portion of ALA is converted into DHA covalently bonded to the 2-acyl position of phosphatidyl choline in plasma [10]. However, the significance of this product in plasma in a single male subject as it applies to the pleotropic properties in brain mediated by the administration of ALA is unknown, raising the possibility that ALA exerts actions of its own. Addition of ALA to cells in culture where metabolism of ALA would not be expected to take place increased neuronal stem cell survival and reduced neuronal cell death in a model of *N*-methyl-d-aspartate (NMDA) receptor-mediated excitotoxicity [3]. In weanling rats made deficient in essential fatty acids, administration of ALA resulted in a reduction in omega-6 polyunsaturated fatty acids such as arachidonic acid suggesting that omega-3 PUFAs exert their effect in brain by inhibiting the desaturation of dihomo-γ-linolenic acid to arachidonic acid. Although ALA was converted to docosahexaenoic acid, there were no differences in the brain levels of eicosapentaenoic acid, a metabolite of ALA [11]. In contrast, administration of ALA in the form of perilla oil to a group of spontaneously hypertensive rats did not change the ratio of unsaturated to saturated phospholipids but there were marked differences in the proportion of omega-3 and omega-6 fatty acids compared with a group of rats administered safflower oil. The most notable difference was a decrease in the proportion of the omega-3 PUFA docosahexaenoate in phospholipids and an increase in the omega-6 PUFAs docosatetraenoic and docosapentaenoic acids compared with the perilla oil group of animals. Importantly, the correct response ratios were higher in the perilla oil group (high ALA) of animals compared with their safflower counterparts in a learning discrimination task [12]. Administration of sunflower oil to rats which is low in ALA resulted in changes at the cellular level. For example, the sodium-ATPase activity in neuronal membranes was reduced by 40% whereas the activity of the 5’ nuclease enzyme was reduced by 20%; these changes were associated with significant learning impairment and neurons were more sensitive to injection of a neurotoxin [13]. These cellular changes may in part be due to alterations in the fluidity within the plasma membrane. ALA has been shown to increase membrane fluidity which may maintain or restore membrane function in undamaged and damaged cells respectively [14]. It has been suggested that the high ratio of omega-6/omega-3 PUFA may be involved in the pathogenesis of many diseases, including cardiovascular disease, cancer inflammatory and automimmune disorders [14]. While many believe that a balance between omega-6/omega-3 PUFA may be important, the “ratio theory” remains controversial. Taken together, ALA has been shown to undergo conversion to docosapentaenoic and docosahexaenoic acids in brain but whether the conversion products are required for the actions of ALA is currently unknown.

## 2. ALA Protects against Animal Models of NMDA Receptor-Mediated Excitotoxicity

Glutamate is the major excitatory neurotransmitter in brain. Paradoxically, the pathophysiology of hypoxic-ischemic neuronal damage in acute and chronic neurodegenerative disorders involves glutamate. The glutamate receptor subtype *N*-methyl-d-asparate (NMDA) plays a major role in neuronal damage [15,16,17,18] and references therein).

In a well-established model of epilepsy induced by kainic acid, ALA treatment, but not other PUFAs or saturated fatty acids, was able to almost completely abolish neuronal cell death in the hippocampal CA1 and CA3 subfields. While other PUFAs exerted neuroprotective efficacy *in vivo*, ALA resulted in the most efficacious and reproducible effect [19]. Surprisingly, the intravenous administration of ALA (500 nmol/kg) significantly increased the hippocampal levels of activated nuclear factor kappaB (NF-κB), a transcription factor, in a time- and concentration-dependent manner [20,21,22]. Furthermore, the increase in activated NF-κB levels in neurons played an essential role in mediating neuroprotection induced by ALA *in vivo* [20] and by subtoxic concentrations of NMDA against NMDA receptor-mediated excitotoxicity *in vitro* [16]. It has been suggested that NF-κB is involved in neuronal plasticity in addition to its well-known role in inflammatory responses [23,24,25,26]. ALA was demonstrated to be neuroprotective in other models of hypoxic-ischemic neuronal injury [27,28,29,30].

## 3. Organophosphate (OP) Nerve Agent-Induced Excitotoxicity and the Limited Availability of Neuroprotective Therapies

Organophosphate (OP) nerve agents are some of the most deadly toxins known to man. The G series class of OP nerve agents includes soman, sarin, cyclosarin, tabun and VX. These agents penetrate the human body through skin, inhalation, and via the bloodstream. The rapidity of symptom onset depends upon the route of nerve agent exposure. Nerve agents inhibit acetylcholinesterase (AChE) quickly and completely and little to no spontaneous reactivation of the enzyme occurs following exposure to sarin, cyclosarin or soman. In the case of severe nerve agent exposure, absorption into the bloodstream occurs quickly and death occurs within minutes of the development of the cholinergic crisis secondary to respiratory and cardiovascular collapse. Absorption of a volatile nerve agent through the skin results in a more deliberate uptake and accumulation of nerve agent in the bloodstream leading to a slower cholinergic crisis [31].

There has been a disturbing resurgence in OP nerve agent use around the world against military and civilian populations by terrorist groups and organizations. The release of sarin in Matsumoto and in the Tokyo subway by a terrorist organization led to the intoxication of thousands of people and nineteen deaths [32,33,34]. The latest incident was in Syria where more than one thousand people, including 426 children, died in the aftermath of sarin deployment last year [35]. Current therapy against exposure to nerve agents targets selective areas within the body to promote overall survival. Atropine, a muscarinic antagonist, reduces the attended copious secretions, bradycardia and gastrointestinal effects. Pralidoxime (2-PAM), an oxime, reactivates acetylcholinesterase molecules that have not undergone aging. After the phosphate moiety on OP nerve agent binds to the serine residue within the active site of AChE to form an ester, a process known as aging occurs whereby there is an internal dealkylation reaction leading to an OP nerve agent-acetylcholinesterase bond that cannot be reactivated by an oxime [36]. The rate of aging is variable, depending on the toxicity of the nerve agent. Acetylcholinesterase will “age” in only two minutes after binding to soman [37].

The underlying mechanism of OP-induced toxicity is the inhibition of AChE which in turn leads to the excessive accumulation of the excitatory neurotransmitter acetylcholine (ACH) within synapses (the cholinergic phase) resulting in a plethora of signs and symptoms including *Status epilepticus*; overactivation of muscarinic receptors results in the generation of seizures and *Status epilepticus* [38,39,40,41,42,43]. Overstimulation of muscarinic receptors by the excessive synaptic levels of acetylcholine and ischemia secondary to the generalized seizures increases the release of glutamate [44,45,46] and γ-aminobutyric acid [GABA] [47,48] disrupting the balance between excitatory and inhibitory input resulting in *Status epilepticus*. The glutamate phase involves glutamate receptors which in turn participate in the propagation and maintenance of nerve agent-induced seizures; the *N*-methyl-d-aspartate (NMDA) glutamate receptor subtype plays a major role in excitotoxic-mediated neuronal cell death in vulnerable brain regions [49,50,51,52].

Prolonged seizures result in brain region-specific neuropathology leading to long-term cognitive and behavioral deficits in animals; long-term cognitive and behavioral deficits have been demonstrated in human survivors [53]. The most profound neuropathology occurs in the amygdala followed by the hippocampus and piriform cortex [21,41]. In fact, the neuropathology can be observed in vulnerable brain regions even when seizures were stopped by benzodiazepines after five minutes [40]. This result alone indicates the danger of exposure to OP nerve agents in survivors. Thus, diazepam, a benzodiazepine, stops/attenuates seizures, but does not prevent the neuropathology [40].

Cognitive and behavioral impairment have been observed years after exposure to OP nerve agents. For example, on the day of the Tokyo subway attack, 5500 individuals that were exposed to sarin gas were evaluated by hospitals. While most complained about minor symptoms, 1000 patients had moderate and 50 patients had severe signs of cholinergic crisis, respectively, associated with low plasma cholinesterase activity and there were 12 deaths [54]. Forty-five percent of victims from the Tokyo subway attack that responded to a survey continued to exhibit symptoms one year after the incident in one study [53]. Post-traumatic stress disorder (PTSD), anxiety, depression, lack of concentration, memory and cognitive deficits, impairment in motor function and coordination as well as structural changes in the right insular cortex and temporal cortex, left hippocampus and loss of white matter in the left temporal area near the insular region on brain MRI [55] develop months to years after exposure to OP nerve agents as well as OP insecticides [56,57,58,59,60,61,62,63]. Importantly, depression and memory impairment are serious and prominent features of exposure to OP nerve agents as well as pesticides [56,61,62,64,65,66]. While depression correlated with PTSD in those exposed to sarin in the Tokyo subway terrorist attack [54], depression is also observed in individuals exposed to OP pesticides [60,61] as well as those individuals exposed to sarin where red blood cell cholinesterase level is between 10% and 40% of control [66] raising the possibility that a cultural difference in Japanese people may have contributed to the depressive symptoms associated with PTSD [54]. Alterations on brain MRI in white as well as gray matter were also observed in Gulf War veterans exposed to OP nerve agents at Khamisiyah [67,68].

Cognitive and behavioral deficits have also been observed in animal models of OP nerve agents. Learning and memory impairments were demonstrated in rodents exposed to OP nerve agents [69,70,71,72,73] including deficits in the Morris water maze and passive avoidance test which are hippocampal-dependent memory tasks [74,75,76]. Deficits in fear-based learning were also shown to be present after exposure to OP nerve agents [77,78]. PTSD and anxiety were demonstrated in rodents exposed to OP nerve agents [78,79,80]. For the first time, our group showed an increased immobility time on the Porsolt forced swim test indicative of a depressed-like state after OP nerve agent exposure [76]. Altogether, the cognitive and behavioral deficits observed in animal models of OP nerve agents replicate the cognitive and behavioral impairment observed in humans that survived exposure to OP nerve agents.

The capability of OP nerve agents to cause mass casualties, the long-term morbidity of individual survivors after exposure to OP nerve agents and the modestly effective antidotal drugs provide the underpinnings for the development of new and more efficacious therapies.

**Figure 1:**
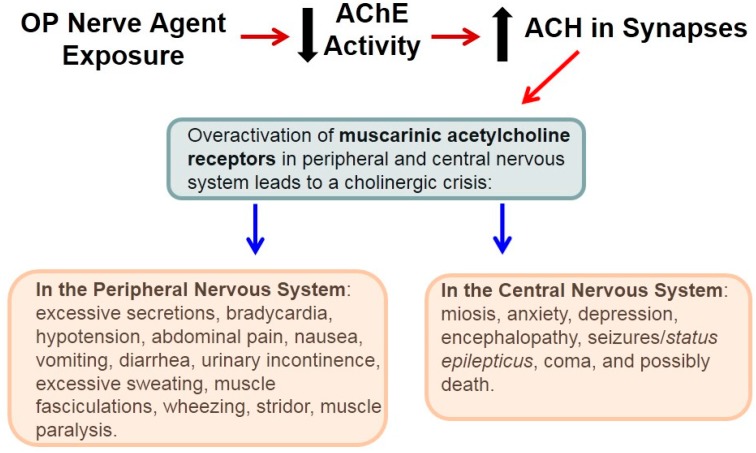
Signs and symptoms after acute intoxication to an OP nerve agent. Exposure to a the chemical warfare agent such as soman results in the inhibition of acetylcholinesterase (AChE) activity leading to excessive accumulation of acetylcholine (ACH) within synapses. These physiological alterations result in the overactivation of muscarinic receptors and a cholinergic crisis in the peripheral and central nervous system.

Exposure to an OP nerve agent results in a plethora of signs and symptoms indicative of a cholinergic crisis in the peripheral and central nervous system (Figure 1) [51]. The rapid increase of acetylcholine that occurs early in soman poisoning is known as the cholinergic phase. During this phase seizures can be blocked with muscarinic receptor antagonists when given immediately after nerve agent exposure. If not controlled, continued seizure activity recruits glutamate and possibly other neurotransmitters to propagate and maintain seizures [45,46,81]. This is the transitional phase with modulation of cholinergic/non-cholinergic systems. During this phase which is referred to as the glutamate phase, seizures cannot be stopped with muscarinic receptor antagonists. Moreover, glutamate can further stimulate the release of acetylcholine contributing to maintenance of the seizures and central nervous system (CNS) neurotoxicity [49,51]. *Status epilepticus* triggers a cascade of effects (overactivation of inotropic glutamate receptors, cytotoxicity, ion imbalance—Ca^2+^ influx—and inflammation) leading to hypoxic-ischemic injury and NMDA receptor-mediated excitotoxicity, the major contributor to neuronal death after OP poisoning.

## 4. Lifesaving Treatment and OP Nerve Agents

The high mortality associated with soman exposure requires treatment with an oxime (HI-6, pralidoxime), a drug that reactivates AChE molecules that have not undergone aging, atropine methyl nitrate, a muscarinic antagonist drug, and diazepam, an anticonvulsant that stops/attenuates seizures [51,82,83,84]. The molecular forms of the oxime and atropine prevent them from crossing the blood-brain barrier so that the neuropathology is reproducible and only controlled by a single drug. Diazepam does cross the blood-brain barrier to stop/attenuate *Status epilepticus* and is used to standardize the model, but diazepam does not prevent the neuropathology [40,85,86,87]. The neuropathology induced by soman is ongoing for at least 3 months [88] that may be due to the development of recurrent seizures after soman exposure [89]. Administration of the oxime, atropine and diazepam increases animal survival and are based upon the current drug use developed for personnel entering areas of nerve agent contamination. These drugs are also given to control animals in order to compare results across all groups ([4,21,41,51,76,82,83,84,90] and references therein). Over the course of the first two to four days after soman exposure, animals lose approximately 20% of their body weight. Supportive care in the form of lactate Ringers is injected subcutaneously to prevent dehydration and wet mash is provided to make it easier for the debilitated animals to eat until they are able to eat and drink on their own [76].

## 5. Vulnerability to OP Nerve Agents Is Brain-Region Specific

The brain regions damaged by soman exposure are the piriform cortex, amygdala, prefrontal/cingulate cortex, hippocampus, caudate/putamen, and thalamus [21,40,41,51,82]. The amygdala and hippocampus are profoundly damaged by soman [4,21,41,76]. The neuronal degeneration induced by OP agents occurs mainly by necrosis, but apoptosis and hybrid forms have been reported in one study [91]. The necrotic process leading to neuronal death is thought to be via NMDA receptor-mediated excitotoxicity after soman poisoning [51,92,93,94].

## 6. The Nutraceutical α-Linolenic Acid Is a Potent Neuroprotective Agent against Soman-Induced Neuropathology

There were no known neuroprotective agents that could be administered systemically against soman-induced neuropathology that weren’t also anticonvulsants, drugs that stop/attenuate *Status epilepticus*, which in turn reduces the brain damage [40,87,95,96,97] or AChE reactivators for those enzyme molecules that were not aged [75,98,99] [NMDA receptor antagonists are not included here due to their adverse effects and the unlikelihood of FDA approval].

A highly sensitive, specific and reproducible method to quantify neuronal degeneration that occurs in vulnerable brain regions after soman exposure is the Fluoro-Jade C staining method. Fluoro-Jade C, a fluorescent ligand that may be the sulfate ester of one of the Fluoro-Jade B components [100], specifically stains degenerating neurons, axons, dendrites and terminals with a very high signal to noise ratio [100]. Cells exhibiting classic morphological features of neurons consisting of a pyramidal shape with a single axon and/or dendrites are identified as neurons. This method can be applied to either fixed ([4,21,76,100,101,102,103,104,105], and references therein) or fresh tissue [106].

**Figure 2:**
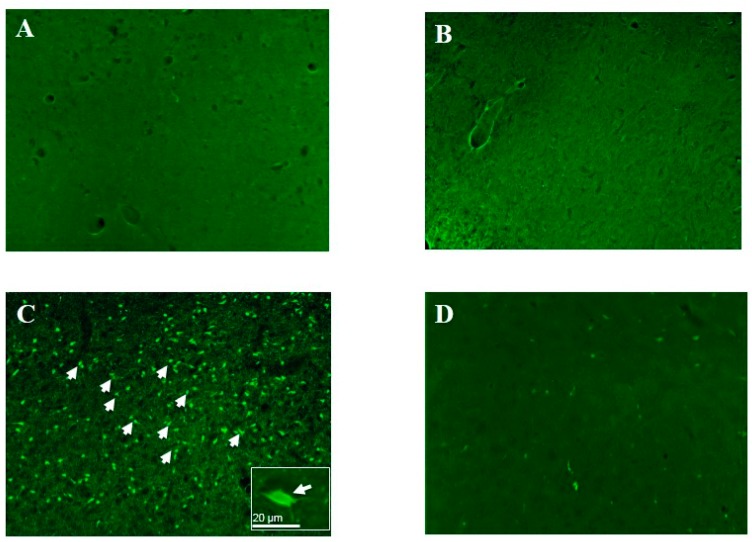
Pretreatment with a single injection of ALA reduces the number of Fluoro-Jade C-positive neurons. Young adult Sprague-Dawley male rats were administered ALA (500 nmol/kg) by intravenous injection 3 days prior to soman exposure or vehicle (0.05% ethanol). All animals were injected with the oxime, HI-6 (125 mg/kg, intraperitoneally [i.p.]) thirty min prior to soman followed by soman (180 μg/kg, subcutaneously [s.c], 1.6 x LD_50_) or saline (control). This dose of soman was chosen because it reproducibly elicits seizures in 100% of the animals tested [21]. Atropine methyl nitrate (2 mg/kg, intramuscularly [i.m.]) is injected 1 min after soman. Rats were allowed to seize for 40 min and then treated with the anticonvulsant diazepam (10 mg/kg, i.m.) to stop/attenuate the *Status epilepticus*. Rats exposed to soman are monitored for seizure activity by the modified Racine scale: Stage 0, no behavioral response; Stage 1, behavioral arrest; Stage 2, oral/facial movements, chewing, head nodding; Stage 3, unilateral/bilateral forelimb clonus without rearing, Straub tail, extended body posture; Stage 4, bilateral forelimb clonus plus rearing; Stage 5, rearing and falling; and Stage 6, full tonic seizures. Stage 6 behavioral seizures were observed 4–8 min after soman exposure and continued until the soman-exposed animals were injected with diazepam (10 mg/kg, i.m.) 40 min after the initiation of *Status epilepticus*. After injection of diazepam, all animals became comatose. Animals were euthanized, brains fixed and post-fixed 24 h after soman exposure. Sections through the basolateral amygdala were obtained from saline/vehicle (**A**); saline/ALA500 (**B**); soman/vehicle (**C**) and soman/ALA500 (**D**) and stained with Fluoro-Jade C as described previously (4, 21, 76). A significant increase in the number of fluoro-Jade C-positive neurons (arrowheads) is observed in the soman/vehicle (**C**) compared with the saline control (**A**). The inset shows the morphology of a typical neuron consisting of a pyramidal shape with three processes.

Representative images using the Fluoro-Jade C staining method after soman exposure in the basolateral amygdala are shown in Figure 2. After exposure to soman, there is a striking increase in the number of Fluoro-Jade C-positive neurons (degenerating neurons) in the basolateral amygdala compared to saline-vehicle (compare Figure 2C with Figure 2A). Administration of a single intravenous injection of ALA (500 nmol/kg) 3 days prior to soman exposure significantly reduced the number of Fluoro-Jade C-positive neurons in the basolateral amygdala compared to saline-ALA500 (compare Figure 2D with Figure 2B) when examined 24 h after soman exposure. ALA not does affect seizure threshold, seizure severity or seizure duration [4]. Previous work showed that a single intravenous injection of ALA administered 30 min after soman also protected neurons in vulnerable brain regions against soman [21]. We also had preliminary data suggesting that the higher ALA dose (500 nmol/kg) was more efficacious than a lower dose (100 nmol/kg) [21]. These findings suggested that ALA may be capable of preventing the primary as well as the secondary neuronal damage triggered by soman, which in turn could reduce the attendant cognitive impairment. Taken together, this was the first demonstration that the nutraceutical ALA exerted a potent neuroprotective effect against soman-induced neuropathology. The next critical issue was to determine whether the reduction in neuronal degeneration improved functional outcome.

## 7. Cognitive Deficits and Neurodegeneration

Alterations in learning, memory and behavior are long-term deleterious effects of OP nerve agent intoxication. Soldiers exposed to OP nerve agents, as occurred during the 1991 Iraq Gulf War [107], civilians in the 1995 Tokyo subway terrorist attack [60,108], and subjects intentionally injected with an OP nerve agent [66] suffer from neurological and neuropsychiatry disorders—namely, cognitive and memory impairments, and depression. Behavioral tests are able to access several learning and memory processes involving the hippocampus, cortex and amygdala in animal models of OP nerve agents. Because the amygdala and hippocampus are severely damaged by OP nerve agents [40,41,84,109,110,111], it is possible that these brain regions play a pivotal role in the long-term cognitive and behavioral deficits reported in rodents and possibly humans after nerve agent exposure.

After exposure to soman, rodents exhibit cognitive deficits in the Morris water maze, a spatial memory task [69,71,72,73], passive avoidance [74,75], active avoidance [112,113] and fear conditioning [77]. The profound hippocampal injury after soman exposure is associated with the poor performance in the Morris water maze [70]. In sharp contrast, no alteration was found in the Morris water maze after an intrahippocampal soman injection that does not induce neurodegeneration indicating that it is the damage that mediates the poor performance [114].

## 8. The Nutraceutical ALA Improves Cognitive Function and Exerts an Anti-Depressant Effect after Soman Exposure *in Vivo*

Because of the profound deficits in standard behavior tasks observed in soman-exposed rats and the significant mortality, we used a dosing schedule that we hypothesized would improve performance on the behavior tasks as well as animal survival. We also administered the α-linolenic acid after soman to reflect a real-life scenario. After exposure to soman or saline, animals were intravenously injected with ALA500 (α-linolenic acid, 500 nmol/kg) or vehicle at 30 min, 3 days and 7 days after soman. ALA500 significantly reduced the soman-induced animal mortality 21 days after soman exposure. For each behavior task, animals were trained prior to being tested. Testing began on day 16 after soman exposure [76]. The open field test was employed because it is a preliminary measure for motor impairment or weakness in rodents [115]. Thus, fifteen days after soman exposure, cohorts of animals exposed to soman or saline and either ALA500 or vehicle were placed in the open field for one hour for training. On the following day, the cohorts were placed back in the open field for their test day. We found no differences across all groups of animals in their locomotor activity. The next behavior task we performed was the rotarod task. Soman-exposed animals spend significantly less time on the rotarod compared with their cohort saline control animals [76]. In sharp contrast, intravenous administration of ALA500 at 30 min, 3 days and 7 days after soman exposure significantly improved motor performance on the rotarod. Depressive behavior was tested next using a modified version of the Porsolt forced swim test, a widely used test to evaluate antidepressant efficacy [116,117]. The Porsolt forced swim test is a model of behavioral despair and a widely accepted test that predicts antidepressant efficacy [117]. Experiments that assess depressive-like behavior had not been yet studied in models of soman exposure in rats. The time spent immobile has been identified as correlating with depressive-like activity and can be reduced by antidepressants drugs [117]. Soman exposure induced a profound effect on the forced swim test immobility time. Rats exposed to soman spent significantly more time immobile compared to saline/vehicle and saline/ALA500-treated groups of animals [76]. The increase in immobility time in the forced swim test after soman exposure cannot be attributed to changes in locomotor performance since no significant differences were apparent in soman-exposed rats in the open-field test compared to respective controls. For the first time, administration of ALA500 at 30 min, 3 days and 7 days after soman effectively reverses the observed behavioral despair in the Porsolt forced swim test. The depressive-like symptoms induced by soman in animals is similar to clinical observations of deployed veterans exposed to nerve agents [118], those individuals exposed to sarin gas in the Tokyo subway [54], volunteers who were exposed to a sarin-like compound where depression was manifested when acetylcholinesterase activity levels were between 10% and 40% [66] as well as the intentional or accidental exposure to OP pesticides [61,62]; in these cases, depression was particularly prominent in women [119]. The observed increase in immobility time in the forced swim test after soman exposure may be a neuropsychiatric consequence of neuronal loss in limbic brain structures, as evidence indicates that mood disorders are characterized by enhanced neurodegeneration [120,121,122]. Structural changes on brain MRI consisting of alterations in white and gray matter have also been observed in soldiers exposed to OP nerve agents during the Gulf War [67,68,123,124]; structural changes were positively correlated with behavior testing [68]. Despite these results, there is some controversy about whether neuronal degeneration or reduced neurogenesis is the fundamental mechanism [125].

Interestingly, a diet restricted in α-linolenic acid reduced brain-derived neurotrophic factor (BDNF) levels in the cortex in mice [126] whereas increased intake of ALA and other polyunsaturated fatty acids increased BDNF protein levels and reduced depressive symptoms in humans and in animals [127,128,129,130,131]. Recently, epigenetic alterations in the elongation of very long-chain fatty acids protein 5 (Elovl5) of polyunsaturated fatty acids were associated depression and suicide risk [132].

The ability of ALA to exert an anti-depressant-like activity in animals exposed to soman may be due to the enhanced protein expression of BDNF in the hippocampus and/or cortex [3,4], two brain regions that are critically involved in adaptive responses. Brain-derived neurotrophic factor is widely expressed in brain [133] and is involved in neuronal maintenance, neuronal survival, learning and memory and neurogenesis [14,134,135,136,137]. It has been shown that direct injection of BDNF into the dentate gyrus or CA3 subfield of the hippocampus exerts an antidepressant effect and the antidepressant-like effect mediated by paroxetine is enhanced in rodent models of depression [138,139]; enhanced BDNF signaling enhances mood [140,141]. Systemic administration of BDNF also produces an antidepressant-like effect [142]. In contrast, genetic deletion of a single allele of the BDNF gene (heterozygous BDNF knockout mice) or conditional deletion of the TrkB gene in neural progenitor cells of mice impairs proliferation and neurogenesis in the dentate gyrus of the hippocampus and leads to loss of antidepressant efficacy [143,144,145]. Importantly, intravenous injection of ALA (500 nmol/kg) on day 1, day 3 and day 7 significantly increased BDNF protein levels in the cortex and hippocampus and exerted an antidepressant-like activity in normal mice [3]. The identical dosing schedule of ALA significantly increased BDNF protein levels in the hippocampus in soman-exposed rats and exerted an anti-depressant-like effect [4,76]. Taken together, exposure to soman results in a depressed-like state as measured by the immobility time in the Porsolt forced swim test. Intravenous administration of ALA at 30 min, 3 days and 7 days after soman exposure animals effectively exerted an antidepressant effect suggesting that the enhanced BDNF levels in the hippocampus contributes at least in part to the observed antidepressant-like activity in soman-exposed animals.

## 9. The Amygdala and Hippocampus, Two Brain Regions Profoundly Damaged by Soman

The amygdala and hippocampus play a critical role in emotional memory and learning and memory respectively [146]. Therefore, it is reasonable to predict that cellular damage in these regions after soman exposure could be associated with functional deficits in some cognitive and behaviors tasks. The hippocampus and amygdala are two limbic brain regions that are required for the passive avoidance test [147,148,149,150,151,152] and they are the same two brain regions that are profoundly damaged by the OP nerve agent soman. In addition, a recent study suggests that cholinergic input particularly nicotinic receptors play an important role in consolidation and retention in the passive avoidance task [153,154]. The significant deficit induced by soman in the passive avoidance task (see above) and the requirement of the amygdala and hippocampus for the successful performance of this test provided a unique opportunity to evaluate the magnitude of ALA to restore cognitive function. The use of the passive avoidance task in soman exposed animals can be referred to as a “stress test” for ALA as the ability of ALA to improve performance was expected to be difficult because the most damage occurs in these two brain regions after soman exposure [41].

Groups of animals were exposed to soman or saline followed the intravenous injection of either ALA500 or vehicle at 30 min, 3 days and 7 days. Cohorts of animals were trained for the passive avoidance task sixteen days after soman exposure. Retention latency was determined 24 h and 5 days later. Retention latency analyzed 24 h after training showed no impairment in soman/vehicle, soman/ALA500 or saline control animals indicating that memory retention occurred in the brain. However, a deficit in memory retention was evidenced by reduced retention latency in the soman/vehicle group of animals when tested five days after training compared to saline/vehicle and saline/ALA500 groups of animals [76]. In contrast, intravenous administration of ALA at 30 min, 3 days and 7 days after soman reversed the reduced retention latency in soman-exposed animals 5 days after soman. These results show that ALA is a highly efficacious therapy against the memory deficits in soman-exposed animals.

As stated above, neuronal loss was associated with the poor performance in the Morris water maze and other behavioral tests. In a similar fashion, neuronal loss in the hippocampus correlated with significant impairments in the passive avoidance task after kainate-induced *Status epilepticus* in rats [155]. Other Pavlovian tasks involving learning and memory in the hippocampus and amygdala such as fear conditioning are also impaired in rats exposed to soman and were associated with neuronal loss and degeneration in the amygdala [77,156]. Significantly, intravenous administration of ALA markedly improved performance on the passive avoidance task five days after training in the soman-exposed rats. Improvement in this task as well as the rotarod and Porsolt forced swim test was associated with a significant reduction in neuronal degeneration 21 days after soman exposure [76]. These data support the idea that ongoing neuroprotection by ALA may mediate in part the significant improvement in cognitive performance.

## 10. OP Nerve Agents, Cognitive Deficits and Neurogenesis

The controversy over whether brain damage or reduced neurogenesis may underlie the cognitive deficits after exposure to a neurotoxin such as soman and the significant reduction in neuronal degeneration after ALA treatment 21 days after soman exposure prompted us to evaluate neurogenesis in this model. Some data indicate that the neurodegenerative process in the piriform cortex, amygdala and hippocampus underlie the pathophysiology of the cognitive deficits [70,71]. However, increasing evidence suggests that reduced neurogenesis may also be a contributory factor to nerve agent-induced long-term cognitive and behavioral disorders; impaired adult neurogenesis in rodents has been shown to be associated with defective spatial and contextual memory [21,73,157,158,159,160].

Over the past decade, it has become apparent that new neurons are still formed in particular regions of the adult brain, but their role in neurodegenerative disorders is not clear. Detection of neurogenesis has been made possible by combining BrdU (5’-bromo-2’-deoxyuridine), a thymidine analogue that is incorporated into DNA undergoing replication, with cell-specific proteins present in the newly generated neurons at different stages of development [161].

Neurogenesis occurs in at least two regions of the adult mammalian brain: the subventricular zone (SVZ) in the lining of the lateral ventricles, and the subgranular zone (SGZ) of the dentate gyrus (DG) in the hippocampus. Neurogenesis has a particular importance in the hippocampus because this brain region is critically involved in learning, memory and mood. The hippocampal DG harbors progenitor cells located in the SGZ which is a thin band of tissue adjacent to the innermost layer of granule neurons [162,163]. The neural progenitor cells located at SGZ give rise to dentate granule cells (DGCs) in the granule cell layer (GCL) where they differentiate into neurons and mature over several weeks [161]. As they mature, a fraction of those newly generated neurons become integrated and form synapses, glutamatergic input from the entorhinal cortex and output to pyramidal cells in the CA3, in a functional hippocampal network [164]. These processes are modulated both positively and negatively by neurotransmitters, hormones, neurotrophic factors, pharmacological agents and environmental factors [137,165,166,167,168]. 

The effect of neurogenesis on animal models of epilepsy induced by various compounds is complex and beyond the scope of this review. In the case of soman, however, there is very little information about the effect of this OP nerve agent on neurogenesis. In one study, mice were injected with soman followed by an oxime and atropine in the absence of diazepam. In this study, the DG cells that were marked only with the proliferation–marker bromodeoxyuridine (BrdU) showed the following changes: a decrease after the first day, an increase at the third day, no changes between day 8 and 30 compared to the control, and a marked reduction in BrdU-positive cells 90 days after the insult, with a very low number of mature neuronal cells double marked with NeuN on day 34 [157]. In another study, double-labeling with BrdU and the immature neuronal marker doublecortin (DCX) showed a decrease in neurogenesis on the 28th day after soman exposure with an associated spatial learning impairment [73].

The participation of newly born neurons in hippocampal processing has been recently discussed in reports showing that changes in neurogenesis are associated with learning performance in hippocampus-dependent learning tasks [169]. Reduced hippocampal neurogenesis has been shown to result in deficits in contextual fear conditioning [159,170,171,172,173], spatial long-term memory [174], trace memories [175] and pattern separation [176]. Reduced neurogenesis has also been implicated in mood disorders, such as depression, but recent studies have raised several controversies [177]. Recent studies using the passive avoidance test have now demonstrated that baseline neurogenesis is required for hippocampal learning and long-term memory formation. Reduced neurogenesis through rapid X-ray ablation causes impairment in performance of the passive avoidance task [178,179].

Conversely, a variety of studies have consistently demonstrated that enhanced neurogenesis plays an important role in exerting the therapeutic efficacy of anti-depressant agents [180] and its potential to affect contextual-memory systems has been increasingly recognized [181,182,183,184]. Consistent with experimental studies, a computational approach has been able to draw a model to understand how hippocampal networks are likely to be selected for encoding information. In the hypothesis of how new neurons affect learning and memory, the new neurons are *added* to the DG network, instead of replacing existing neurons. This addition could effectively encode and retrieve new memories in the network without interfering with old memories [185]. However, new research has proposed that the continuous integration of new neurons may affect memories already stored in these circuits by competing with existing cells for inputs and outputs when examined over longer times [186].

We demonstrated previously that the intravenous administration of ALA on day 1, day 3 and on day 7 enhanced neurogenesis and increased the number of mature neurons in the subgranular zone of the hippocampus in naïve mice [3]. However, whether this dosing schedule could increase neurogenesis in a damaged brain and whether the hypothesized enhanced neurogenesis by ALA was involved in the improvement in the cognitive performance in soman-exposed animals were unknown. If neurogenesis was increased by ALA, what signaling mechanisms were involved?

The mammalian target of rapapmycin, a serine/threonine kinase that plays a crucial role in the regulation of cellular proliferation and growth. Intravenous administration of ALA at 30 min, 3 days and 7 days after soman exposure significantly increases the endogenous expression of mature BDNF protein levels in the hippocampus [4]. BDNF is released via the constitutive or activation-dependent pathway into the extracellular milieu. When mature BDNF binds to and activates full-length TrkB, its cognate receptor, TrkB receptor monomers dimerize, which increases the catalytic activity of the intracellular domain of the intrinsic tyrosine kinase. This enables the phosphorylation of tyrosine residues inside the activation loop and subsequently the autophosphorylation of tyrosine residues situated outside of the activation loop. The next step is the activation and recruitment of partner proteins and adaptors that lead to the activation of three main intracellular signaling pathways: [1] the phospholipase Cγ (PLCγ) pathway, which leads to activation of protein kinase C; [2] the mitogen-activated protein kinase (MAPK) or extracellular signal-regulated kinase (ERK) pathway, which activates several downstream effectors; and [3] the phosphatidylinositol 3-kinase (PI3K) pathway, that activates the serine/threonine kinase Akt. Both MAPK and PI3K play crucial roles in neuronal survival, protein-synthesis dependent plasticity and neurogenesis [134,187,188,189,190,191,192,193,194,195,196]. A pathway regulated by nutrients and growth factors *i.e.*, BDNF is the mammalian target of rapamycin complex 1 (mTORC1). Once Akt is fully activated via phosphorylation of Ser^473^ and Thr^308^, this serine-threonine kinase activates mTORC1 [197,198].

Mammalian target of rapamycin is a large (~289 kDa) serine-threonine kinase that exists in two distinct heteromeric protein complexes referred to as mTOR complex 1 (mTORC1) and mTOR complex 2 (mTORC2). The mTORC1 is sensitive to the selective inhibitor rapamycin and it is activated by phosphorylation of serine^2448^ in the canonical PI3K-Akt pathway after growth factor stimulation [199]. Rapamycin does not directly inhibit mTOR kinase activity; instead, it binds to the immunophilin FK506-binding protein 1A, (FKBP12) and disrupts the mTOR-RAPTOR interaction, e.g., the complex of mTORC1. By contrast, mTORC2 is resistant to rapamycin, and recent studies have suggested that mTORC2 may phosphorylate Akt at Ser^473^ [200,201] but its mechanisms in brain plasticity and neurogenesis are not well understood.

It has been demonstrated that BDNF is required for baseline neurogenesis in the hippocampus [137]. Activation of TrkB via BDNF activates the PI3K pathway and downstream activation of Akt which in turn would activate mTORC1. The activation of mTOR signaling by neurotrophins increases the translation of synaptic proteins, which is essential for synaptic plasticity, by two mechanisms: First, mTORC1 phosphorylates and inactivates the eIF4E-binding protein (4E-BPs), facilitating translation initiation by releasing the inhibition of eukaryotic initiation factor 4E (eIF4E), which is a crucial initiation factor in cap-dependent translation. The association of 4E-BPs with eIF-4E inhibits the ability of eIF-4E to associate with eIF-4G and initiate translation. Second, mTORC1 leads to the activation of p70 S6 kinase, an enzyme that controls translation at a number of levels, including synthesis of the S6 ribosomal subunit, phosphorylation of RNA helicase cofactor eIF4A, and inhibition of eukaryotic elongation factor 2 (eEF2) kinase [202]. The p70 S6 kinase and 4E-BP are also regulated by MAPK/ERK to control protein-synthesis dependent plasticity [203], and p70 S6 can further phosphorylate mTOR setting up an autoregulatory mechanism. Protein synthesis-dependent synaptic plasticity strengthens the neuronal connection and would be expected to regulate memory storage in the brain. On the other hand, excessive protein synthesis results in behavioral deficits, instead of improving neuroplasticity, suggesting a temporal window to where mTOR should be carefully modulated [202]. Recently, one study found that rather than just increasing translation of new proteins for synaptic plasticity, mTORC1 activation leads to the induction of genes encoding the enzymes of glycolysis, pentose phosphate pathway and lipid sterol biosynthesis, generating the building blocks for anabolic cell growth [204]. This process could help the development and maturation of new neurons in adult neurogenesis and also cellular repair. The newly generated neurons integrate multiple signals into local circuitry of the hippocampus and several lines of evidence have revealed the importance of these newborn neurons in the acquisition and retention of memories [137,174,184,205] and mood control [206].

We set out to address whether intravenous administration of ALA at 30 min, 3 days and 7 days after soman exposure increased neurogenesis in the subgranular zone (SGZ) of the hippocampus. We first confirmed that this dosing schedule increases the endogenous expression of mature BDNF levels in the hippocampus. We then tested the hypothesis that the increased expression of mature BDNF levels in the hippocampus leads to an increase in activated Akt and activated mTORC1. We showed that an increase in activated Akt and mTORC1 only occurred in those groups of animals intravenously administered ALA500. We did not find an increase in activated Akt or mTORC1 in the hippocampus from animals exposed to soman-vehicle despite the fact that the endogenous expression of mature BDNF levels increased in the hippocampus in this group of animals [4]. One possible explanation for the differential effect was that the mature BDNF was expressed in different cells. To address this possibility, we carried out an immunohistochemical analysis using an antibody against the mature neuronal marker, NeuN, and an antibody against mature BDNF. Cells co-localizing NeuN and mature BDNF were identified as neurons. In the case of animals exposed to soman or saline followed by ALA, we found an increase in the number of cells where NeuN co-localized with mature BDNF identified as neurons in the dentate gyrus compared to saline-vehicle and soman-vehicle-exposed animals. These results suggest that the neuronal expression of mature BDNF in the hippocampus is induced by ALA. In sharp contrast, the neurotoxin soman increases the endogensous expression of mature BDNF in non-neuronal cells [4]. We then hypothesized that this dosing schedule of ALA would increase neurogenesis in the SVZ in animals exposed to soman. In this experiment, groups of animals were exposed to soman or saline followed by ALA or vehicle at 30 min, 3 days and 7 days. Another group of animals was also injected with the long-term inhibitor of mTORC1, rapamycin. We showed that the intravenous administration of ALA significantly increased neurogenesis (doublecortin-positive) 10 days after saline and increased the number of mature neurons at 31 days confirming our previous findings [3]. Moreover, we found that the intravenous administration of ALA significantly increased neurogenesis in the group of animals exposed to soman 10 days after soman exposure. We also found a significant increase in the number of mature neurons in the SVZ on day 31 after soman exposure. We showed that the mTORC1 inhibitor, rapamycin, completely blocked the ALA-induced neurogenesis in the saline control animals and in the group of animals that were exposed to soman [4] suggesting that the activation of mTORC1 was critical for neurogenesis. The next step was to determine whether the increase in neurogenesis was involved in the ALA-mediated enhancement in cognitive performance. The passive avoidance was selected as the behavior measure to determine the role of neurogenesis because this task requires the amygdala and hippocampus and both brain regions are profoundly damaged by soman. For this experiment, groups of animals were exposed to soman or saline followed by either ALA or vehicle. Some groups of animals were also injected with rapamycin over a 7 day period. All groups of animals were trained in the passive avoidance task 16 days after soman exposure and tested 24 h and 5 days later. We confirmed that animals exposed to soman only showed a significant deficit in retention latency 5 days after training; no deficit was observed 24 h after training across all groups of animals [4,76]. Intravenous administration of ALA at 30 min, 3 days and 7 days after soman resulted in a striking improvement in retention latency in soman-exposed animals 5 days after training. Administration of rapamycin, however, completely blocked the ALA-induced improvement in retention latency. Because rapamycin also blocked the ALA-induced increase in neurogenesis, our results suggest that enhanced neurogenesis induced by ALA plays a critical role in the ALA-induced improvement in retention latency in the passive avoidance via an mTORC1-mediated mechanism. The downstream mTORC1 pathway is likely activated by the increase in activated Akt via a BDNF-TrkB-mediated mechanism.

## 11. ALA Induces Diverse and Mechanistically Distinct Endogenous Neuroprotective Pathways

**Figure 3:**
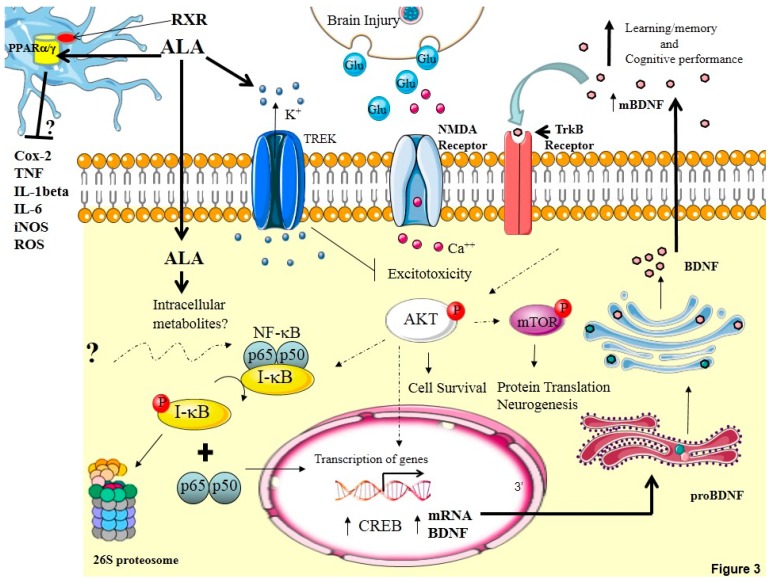
Overview of possible mechanisms involved in ALA-mediated neurorestoration. Injury to the brain results in the excessive release of glutamate (Glu) leading to the overactivation of NMDA receptors and neuronal cell death. Administration of ALA results in the formation of lipid rafts, an increase in activated nuclear factor kappaB (NF-κB) levels and cyclic AMP response element binding protein (CREB) [216] and enhanced levels of BDNF mRNA and protein in the hippocampus. Release of mature BDNF into the extracellular milieu results in the rapid activation of TrkB receptors and NMDA receptors to promote neuronal survival and enhance neurotransmission [217]. Activation of TrkB leads to the downstream activation of Akt which in turn activates mTORC1 to increase neurogenesis, differentiation and an increase in the number of mature neurons. The increase in mature neurons can integrate into and enhance functional neuronal networks which in turn enhances cognitive and behavioral outcome. It is also possible that ALA binds to the peroxisome proliferator activated receptors gamma (PPARγ) and/or peroxisome proliferator activated receptors alpha (PPARα) along with the retinoic acid receptor (RXR) in the nuclei of microglia to reduce the release of pro-inflammatory mediators such as interleukin-1 beta (IL-1β), interleukin-6 (IL-6), the inducible form of nitric oxide (iNOS), tumor necrosis factor (TNF), cyclo-oxygenase-2 (COX-2) and reactive oxygen species (ROS) possibly by reducing the activity of NF-κB.

To date, the overall summary of the fundamental mechanisms that appear to be involved in protecting the hippocampus against soman-induced neuropathology by ALA is shown in Figure 3. After the brain injury, intravenous administration of ALA is taken up into the central nervous system. ALA has been shown to activate TWIK-related (TREK)-1 channels, two-pore rectifier potassium channels [207]. Activation of the TREK-1 background potassium channels would be expected to hyperpolarize the membrane which in turn would reduce the release of glutamate and NMDA receptor-mediated excitotoxicity. ALA has also been shown to induce lipid rafts that along with some NMDA receptors and TrkB would markedly enhance signaling efficiency [14,208,209,210,211]. ALA uptake into the membrane followed by release intracellularly or direct uptake into the intracellular compartment and/or of its long chain metabolites may mediate a diverse and mechanistically distinct complement of molecular and cellular events to improve neuronal survival and restore function.

For example, ALA has been shown to increase BDNF mRNA and protein levels in the hippocampus, in cultured hippocampal neurons and in neural stem cells [3]. The increase in BDNF mRNA may be mediated in part by the well-established increase in activated NF-κB levels in the hippocampus [20,21]. Activation of NF-κB via the canonical pathway involves the phosphorylation of the inhibitor, IκB at Ser^32^. Once phosphorylated mainly by Iκ kinase, IκB dissociates from NF-κB and is degraded by the 26 s proteasome (Figure 3). Activated NF-κB translocates to the nucleus where it binds to and activates gene transcription as long as the p65 subunit is one of the dimer subunits. The increase in the endogenous expression of mature BDNF protein levels by ALA stimulates neurogenesis that would be expected to exert multiple effects. Research activities focusing on neural stem cells have shown that promoting their proliferation or grafting/infusing them by different routes into the brain leads to neurological improvement in different brain disease models [212,213,214,215]. This benefit does not come only by their capacity to replace lost neurons. They can also trigger several other mechanisms, such as the induction of survival-promoting neurotrophic factors or promote the restoration of synaptic transmitter release function by integrating into existing synaptic networks, and thus improve the functional circuitry [164]. Administration of three injections of α-linolenic acid promote neural stem cell proliferation, synaptogenesis and synaptic function [3]. It is worth noting that neural stem cells can induce the transcription of protective factors, such as BDNF, which would be expected to modify the ischemic environment and promote neuroprotection. In the scenario where administration of ALA induces the formation of lipid rafts, NMDA receptors and TrkB would be spatially and temporally close together leading to enhanced functional efficacy. This in turn would lead to a rapid activation of TrkB receptors and the signal transduction pathway resulting in activated Akt which in turn activates mTORC1. The activation of mTORC1 appears to be an essential requirement for ALA-induced neurogenesis.

Activation of mTORC1 phosphorylates and inactivates the eIF4E-binding protein (4E-BPs), facilitating translation initiation by releasing the inhibition of eukaryotic initiation factor 4E (eIF4E), which is a crucial initiation factor in cap-dependent translation and activates the p70 S6 kinase, an enzyme that controls translation at a number of levels. This machinery is likely critically involved in the increase in newborn neurons, the differentiation process leading to integration and new synapse formation within neuronal networks involved in learning and memory in the CA3 subfield of the hippocampus. We propose that ALA may exert an anti-inflammatory effect after brain injury. This anti-inflammatory effect may be on microglia, the resident macrophages of the brain and spinal cord. Resting microglia are thought to develop into primed microglia after brain injury occurs and along with peripheral humoral pro-inflammatory mediators such as IL-1β, IL-6, and/or chemokine ligand 1 (CXCL-1) develop into activated microglia. Activated microglia release pro-inflammatory cytokines and the ongoing neuroinflammation is thought to result in the loss of neuronal integrity and neuronal cell death. The peroxisome proliferator-activated receptors (PPAR) are a family of transcription factors that regulate gene expression and act by forming heterodimers with the retinoic-X-receptor [218]. PPARα and PPARγ receptors modulate the expression of inflammatory genes [219,220]. We hypothesize that ALA binds to PPARγ or PPARα to reduce the synthesis and/or release of pro-inflammatory cytokines from resident microglia possibly by reducing the activity of NF-κB after brain injury [221] (Figure 3). Taken together, ALA targets multiple pathways in brain to promote neuronal survival and improve cognitive function after brain injury. These fundamental mechanisms may be vital to protect against other acute and chronic injuries in the central nervous system that involve NMDA receptor-mediated excitotoxicity.

## 12. Conclusions

In summary, ALA is a nutraceutical with a very wide safety margin. The pleiotropic properties activate transcriptional and translational programs in brain to promote neuronal survival and improve cognitive function but the optimum dose to mediate these effects in humans will require additional studies. The diverse and mechanistically distinct pathways activated by ALA serve as a blueprint for restoring brain function and as such may be efficacious against other acute and chronic neurodegenerative disorders.
